# Genetic Enhancement of Cereals Using Genomic Resources for Nutritional Food Security

**DOI:** 10.3390/genes14091770

**Published:** 2023-09-07

**Authors:** Neeraj Chaudhary, Romesh Kumar Salgotra, Bhagirath Singh Chauhan

**Affiliations:** 1 School of Biotechnology, Sher-e-Kashmir University of Agricultural Sciences and Technology of Jammu, Chatha, Jammu 180009, Jammu and Kashmir, India; nirs.micro@gmail.com (N.C.); rks_2959@rediffmail.com (R.K.S.); 2 Queensland Alliance for Agriculture and Food Innovation (QAAFI), The University of Queensland, Gatton, QLD 4343, Australia

**Keywords:** genomic resources, genetic improvement, cereal crops, food security

## Abstract

Advances in genomics resources have facilitated the evolution of cereal crops with enhanced yield, improved nutritional values, and heightened resistance to various biotic and abiotic stresses. Genomic approaches present a promising avenue for the development of high-yielding varieties, thereby ensuring food and nutritional security. Significant improvements have been made within the omics domain, specifically in genomics, transcriptomics, and proteomics. The advent of Next-Generation Sequencing (NGS) techniques has yielded an immense volume of data, accompanied by substantial progress in bioinformatic tools for proficient analysis. The synergy between genomics and computational tools has been acknowledged as pivotal for unravelling the intricate mechanisms governing genome-wide gene regulation. Within this review, the essential genomic resources are delineated, and their harmonization in the enhancement of cereal crop varieties is expounded upon, with a paramount focus on fulfilling the nutritional requisites of humankind. Furthermore, an encompassing compendium of the available genomic resources for cereal crops is presented, accompanied by an elucidation of their judicious utilization in the advancement of crop attributes.

## 1. Introduction

The basic requisites furnished by food encompass proteins, minerals, carbohydrates, fiber, vitamins, lipids (macronutrients), as well as essential micronutrients, all of which furnish the energy imperative for bodily growth, function, and defense [1]. Belonging to the Poaceae family, all cereal crop species share this familial lineage, making it the fourth largest family of flowering plants [2]. The principal cereal crops, wheat, rice, maize, sorghum, finger millet, foxtail millet, barley, and other such counterparts, stand as pivotal sources of nourishment worldwide [3]. These grain cereals form the cornerstone of staple diets across many regions, and their utility extends beyond sustenance to encompass starch, animal feed, oils, sugar, and processed consumables, such as malts and alcoholic beverages. An impressive 50% of global calories stem from maize and wheat, while regions in Africa and Asia prominently rely on grains like sorghum and millets. As the global populace burgeons and standards of living surge, the demand for ample, high-quality, and diversified sustenance escalates, rendering the task of feeding an anticipated population of 10 billion by 2050 a formidable challenge.

The impending prospect of nourishing 10 billion individuals by 2050 looms as one of agriculture’s paramount challenges [4]. Concurrently, the specter of climate change further imperils food security, predicting heightened severity in the years ahead. Consequently, the augmentation of cereal crops to manifest enhanced productivity and climate resilience is imperative. Unveiling the genetic underpinnings of these traits assumes paramount importance, and herein lies the crux and promise of genetic and genomic strategies. These approaches are instrumental in delineating the genetic determinants responsible for these traits, further enabling their characterization and targeted integration into trait enhancement initiatives. Hence, genetics and genomics emerge as pivotal tools for dissecting the intricate tapestry of traits encompassing growth, development, and responses to environmental pressures. Unquestionably, the cereal research community has harnessed these tools to investigate crucial attributes inherent in cereal crops.

Anticipating that the world’s population will swell to around 11.2 billion by 2100 necessitates not only a surge in food production but also the establishment of secure and sustainable food systems, all while leveraging finite natural resources [5,6,7,8,9,10,11,12]. This challenge is intensified by the dwindling expanse of cultivable land, a consequence of industrialization and burgeoning infrastructure.

Cereal crops collectively constitute edible seed grains for global consumption, offering vital dietary supplements, such as vitamin E, vitamin B, magnesium, and zinc [13]. Among these, rice, wheat, and maize command a staggering 90% share of global cereal production, leaving the remaining 10% to encompass minor cereals like millets, barley, oats, and rye [14]. The advent of the Green Revolution in the 1960s bore witness to escalated yields of the two primary cereals, wheat and rice, curtailing food grain scarcities [15]. Over time, these predominant varieties were further optimized for enhanced yield and other agronomic traits, resulting in sustainable yields that could cater to growing nutritional demands. However, a protracted disregard for the nutritional components of these crops left a substantial void, ultimately fostering malnutrition in approximately one out of every three individuals worldwide [16]. Although traditional plant breeding played a pivotal role in improving cereal traits, addressing intricate attributes like enhanced selenium uptake in wheat to counter abiotic stresses, such as drought and heat tolerance, a noticeable gap remained in nutritional richness. Thus, the imperative to bridge this nutritional divide and ensure sustainable food security underscores the necessity of fortifying cereals using advanced genomic resources for genetic manipulations of major cereal crops.

The practice of fortifying cereal crops with key nutrients, such as vitamins (B, C, D, and E), iron, β-carotene, and zinc, has gained prominence as a strategy for augmenting their nutritional value [17]. Instances of successful nutritional enhancement employing genomic techniques and resources abound, encompassing endeavors, such as elevating the oligosaccharide and polysaccharide levels, enriching the iron content, boosting the vitamin E concentrations in maize, and engendering rice varieties replete with β-carotene and iron [18,19,20,21].

As the basis of future crop breeding, genetic resources stand as pivotal to future food security. Contemporary methods for germplasm characterization and assessment have yielded commendable results in managing crop genetic resources effectively. In parallel, the utilization of genomic resources and specialized germplasm subsets, including mini-core collections and reference sets, facilitates the identification of trait-specific germplasm, trait mapping, and allele mining, not only for resistance to diverse biotic and abiotic pressures, but also for beneficial agro-morphological traits.

The revolution in cereal genomics was inaugurated by the unveiling of draft genome sequences for rice, subsequently followed by sorghum, maize, pearl millet, barley, and wheat [22,23,24,25,26,27,28]. This repository of genome sequences, accompanied by an array of genomic resources, advanced mapping populations, and panels, has propelled genomics-assisted breeding within the realm of cereals [29]. These advancements have significantly advanced the exploration of the gene function vis-à-vis the target phenotypes.

**Figure 1:**
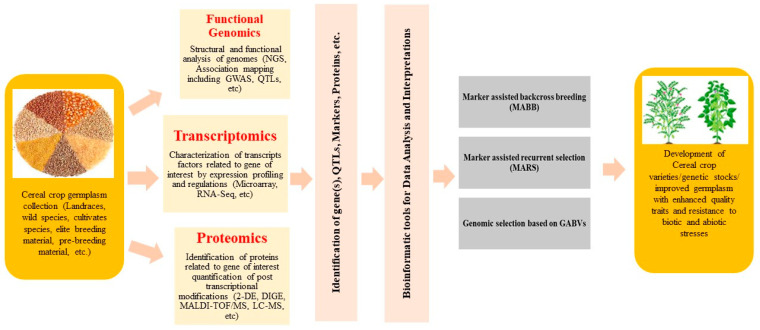
Different genomic approaches used for cereal crop improvements.

Within the annals of botanical knowledge, an abundance of research material delves into the molecular and cellular mechanisms of rice, a model plant for cereal counterparts [30]. The intricacies of signal responses to sundry stresses in crops often hinge upon gene regulation. In this context, contemporary genomic resources, including molecular markers, whole-genome sequences, sequencing-based trait mapping, and germplasm sequencing, facilitate the exploration of gene regulation at the molecular level [31]. Despite tremendous leaps in genomics, avenues, such as transcriptomic and proteomic profiling, remain to be explored fully. The creation of novel crops endowed with enhanced resistance to diverse biotic and abiotic stresses, as well as other nutritional traits, stands as an essential pursuit. A wealth of stress-responsive genes is activated, engendering proteins that orchestrate physiological and biochemical pathways integral to stress tolerance [30,32]. Thus, the implication of genomic approaches with the ambit of systems biology augments our comprehension of intricate mechanisms and charts a course for future endeavors [33,34,35,36]. This review endeavors to elucidate recent strides in functional genomics, transcriptomic analyses, and proteomic investigations, shedding light on the mechanisms orchestrating stress tolerance and nutritional enhancement within diverse cereal crops. A synthesis of the transcriptomic, proteomic, and functional genomic approaches pertinent to crop advancement is succinctly portrayed (Figure 1).

## 2. Genomic Resources

In the pursuit of substantial advancements across various crop species, genomic tools have emerged as the cornerstone, offering essential inputs to breeding endeavors. A pivotal role is played by genomic resources, including linkage and genetic maps, molecular markers, and sequence data, in enhancing molecular breeding programs [37]. As the scientific landscape ascends to new heights, propelled by genomic paradigms and Next-Generation Sequencing (NGS), the analysis of genomes has reached unprecedented dimensions. NGS has permeated diverse domains, encompassing whole-genome sequencing (WGS), whole-genome resequencing (WGRS), de novo sequencing, transcriptomic analyses, genotyping-by-sequencing (GBS), and epigenetic research. These resources have culminated in the creation of several PCR-based markers. Among these markers, namely inter-simple sequence repeats (ISSRs), simple sequence repeats (SSRs), single-nucleotide polymorphisms (SNPs), and diversity array technologies (DArTs), are frequently harnessed to foster crop improvement [38]. While SSR markers find frequent utility in crop breeding [39], ISSRs excel in gauging genetic variation, conducting diversity analyses, and facilitating the molecular breeding of crop plants, owing to their elevated polymorphism levels. Dominating the marker landscape are SNPs, cherished for their facile availability, high-performance genotyping capabilities, and cost effectiveness [25]. The DArT marker system has, notably, surfaced as a robust tool for uncovering genetic diversity within germplasm collections and holds promise in molecular breeding studies [40]. The confluence of these resources expedites the identification of genes/QTLs and paves a well-defined trajectory toward molecular breeding, bolstering crop productivity and ensuring food security [25]. It is worth noting that the resolution of genetic variation closely aligns with the number of polymorphisms detected through the marker system [41].

**Figure 2:**
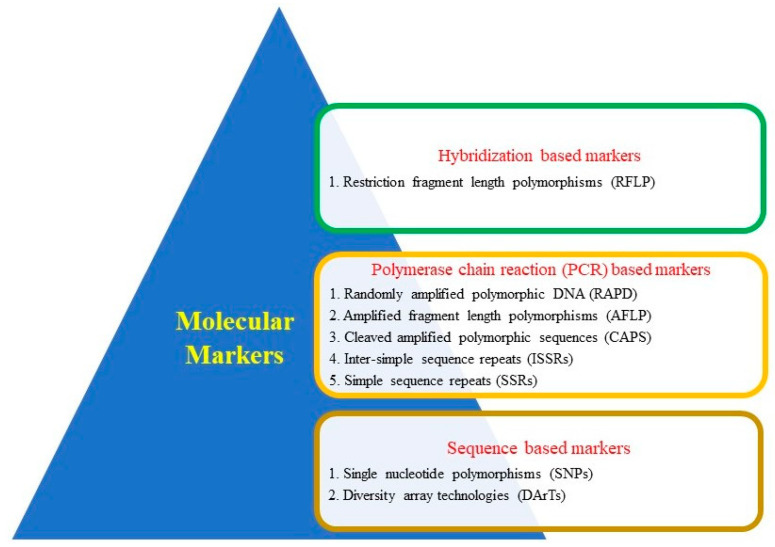
Molecular markers used in MAS for cereal crop improvements.

Historically, the bulk of genomic and molecular research has concentrated on cereal crops, driven by the imperative of global food production [31]. However, the proliferation of NGS techniques has broadened the scope to encompass other crops, illuminating their capacities and prominence. Novel NGS platforms have facilitated the generation of expansive sequence data, giving rise to diverse genomic resources, such as restriction fragment length polymorphisms (RFLP) [42], randomly amplified polymorphic DNA (RAPD) [43], SSRs [44], cleaved amplified polymorphic sequences (CAPS) [45,46], amplified fragment length polymorphisms (AFLP) [47], SNPs [48], and DArT markers [49] (Figure 2). These molecular markers prove instrumental in diverse molecular studies, spanning genetic map construction, indirect selection, and the introgression or pyramiding of quantitative trait loci (QTLs) or genes into elite varieties via molecular breeding strategies like marker-assisted selection (MAS), marker-assisted backcross breeding (MABC), and genomic selection (GS). Notably, genome-wide association studies (GWAS), SNP arrays, and transcriptomic, metagenomic, epigenomic, and gene expression data have accelerated breeding cycles across multiple crops [50,51].

With the advent of high-throughput NGS techniques, genomics has empowered biotechnologists to sequence the genomes of nearly all major cereal crops, enabling the identification of genes/QTLs and facilitating the incorporation of desirable traits into diverse crops [52]. Consequently, researchers are inclined to explore gene upregulation/downregulation and analyze global shifts in gene expression by pinpointing corresponding RNAs. Within transcriptomics, the panorama of gene expression dynamics and functionality is unveiled. The realm of transcriptomics, replete with diverse coding and non-coding RNA molecules, bears witness to remarkable complexity [53]. Thanks to cost-effective, Sanger, and high-throughput NGS technologies, the field of transcriptomics has undergone a paradigm shift, post the completion of genome sequencing projects across various crops (Table 1) [30]. A plethora of sequence data has been generated by the sequencing of important cereal crops. This surge in knowledge has paved the way for downstream investigations in the domains of post-transcriptional and post-translational events [30]. Transcriptomic studies adopt a multitude of techniques, including microarrays, expressed sequence tags (ESTs), serial analysis of gene expression (SAGE), cap analysis of gene expression (CAGE), RNA-Seq/NGS, and de novo assembly. The microarray technique, reliant on hybridization, presents an economical and high-throughput approach, and played a pivotal role in pioneering transcriptomic research [54]. Utilizing a microarray, a DNA chip arrays multiple DNA fragments (probes/reporters) on small glass slides, offering an automated platform for parallel DNA sequence analysis. Thousands of labeled spots containing single-strand DNA/RNA molecules, labeled with fluorescent dye, populate the microarray glass slide. Upon introduction of a solution containing ssDNA or RNA fragments (targets), hybridization transpires, yielding fluorescence. This fluorescence is subsequently scanned and analyzed, furnishing data for further exploration. Presently, two primary microarray types have gained prominence: short oligonucleotide-based and cDNA-based microarrays. The application of DNA microarrays encompasses gene expression profiling, comparative genomic hybridization, SNP detection, and the detection of alternative splicing [55].

The domain of microarray study presents both quantity and quality challenges in regard to data acquisition. Degraded mRNAs can yield false microarray data, and sensitivity to the cross-hybridization of probes with non-target sequences can generate misleading outcomes [56]. The presence of high background noise due to sequence similarity can further complicate result determination. The specificity of probes in DNA microarrays is also influenced by their lengths, with longer probes exhibiting reduced specificity [57]. Additionally, microarray techniques exhibit a limited dynamic range of detection due to both background and saturation signals, necessitating prior knowledge of the sequences under examination [58,59].

Subsequent to microarray techniques, sequence-based methodologies emerged to provide a more comprehensive elucidation of the transcriptome by directly determining the transcript sequences. Complementary DNA (cDNA) libraries are constructed from isolated mRNAs in a manner that allows identification of the 5′ and 3′ ends of the message through sequencing the vector–insert joins in the plasmid. This approach, such as expressed sequence tags (ESTs) generation, facilitates the rapid and easy discovery of new genes, crucial for functional genomics and understanding the molecular mechanisms underpinning sustainable agricultural production [59,60]. However, limitations include the detection of predominantly abundant transcripts, relatively low throughput, and the inability to quantify transcripts [61]. The NCBI’s dbEST stands as a specialized division of GenBank, encompassing publicly available EST collections from various organisms [62].

To surmount the limitations posed by ESTs, tag-based methods such as serial analysis of gene expression (SAGE) and cap analysis of gene expression (CAGE) were developed. SAGE quantitatively estimates mRNA expression levels by measuring short (10–14 mer) sequences of transcribed messages, using them to infer specific transcripts. SAGE aids in detecting and quantifying gene activities in desired cells, providing insights into discovering novel genes. CAGE, conversely, collects 21 bp tags from the 5′ end of cDNAs to ascertain the 5′ end of transcripts and promoter locations [63]. While these techniques yield improved results, challenges persist, including splice isoforms, the inability to discover new genes, the requirement for a significant amount of RNA, labor-intensive procedures, and high sequencing costs. Nonetheless, the advent of high-throughput Next-Generation Sequencing (NGS) technologies, notably RNA sequencing (RNA-Seq), has effectively addressed these limitations, revealing transcriptomes with heightened sensitivity and accuracy [64,65].

RNA-Seq, a form of high-throughput NGS, sequences the RNA of a species that precisely determines each transcript, achieving remarkable sequencing depth [53]. This technique has surmounted previous limitations, elucidating molecular functions through single-cell gene expression and aiding in the identification of novel genes/QTLs [53,59]. NGS has elevated the scale of transcriptome sequencing, broadened sequence-based marker resources, and enabled de novo assembly for crop development, all at a reduced cost compared to phenotyping [66]. This approach also affords a high-resolution view of transcription, alternative splicing, and allele-specific expression. However, RNA-Seq analysis introduces computational challenges unique to this type of sequencing-based investigation [67].

The generation of diverse genomic resources through various technologies has precipitated the genomic revolution [31]. The application of these genomic resources accelerates breeding and other crop improvement programs. These resources furnish the foundation for the efficient utilization of genetic resources in the enhancement of cereals and other crops. For genetic improvement, aside from sequencing data, an array of genetic resources and biparental populations like doubled haploids (DHs), F2 or F2-derived F3 populations, recombinant inbred lines (RILs), backcrosses, near-isogenic lines (NILs), nested association mapping (NAM), and multiparent advanced generation inter-cross (MAGIC) populations are essential for linking gene(s)/QTLs to specific traits. The identified marker–trait associations can then be harnessed for crop enhancement or development using marker-assisted recurrent selection (MARS), marker-assisted backcross breeding (MABB), and genomic selection (GS) approaches [37,68].

**Table 1:** Important cereal crops sequenced using Sanger and NGS technologies.

Crop	Botanical Name	Genome Size (Mb) and Sequencing Method	Reference
Rice	*Oryza sativa* ssp. *japonica* (Nipponbare) *Oryza sativa* ssp. *japonica* (Nipponbare) *Oryza sativa* ssp. *Indica*	(i) 420 Mb; Sanger, Whole-Genome Sequencing (WGS) (ii) 389 Mb; Sanger, Bacterial Artificial Chromosome (BAC-by-BAC) (iii) 466 Sanger, Whole-Genome Sequencing (WGS)	[22] [69] [70]
Maize	*Zea mays* (Palomero Toluqueno) (popcorn) *Zea mays* (B73)	(i) 2100 Mb; Sanger, Whole-Genome Sequencing (WGS) (ii) 2300 Mb; Sanger, Bacterial Artificial Chromosome (BAC-by-BAC)	[71] [24]
Sorghum	*Sorghum bicolor* (L.) Moench	730 Mb; Sanger, Whole-Genome Sequencing (WGS)	[23,72]
Foxtail millet	*Setaria italica*	515 Mb; Illumina, Whole-Genome Sequencing (WGS)	[73]
Bread wheat	*Triticum aestivum*	17,000 Mb; 454, Whole-Genome Sequencing (WGS)	[74]
Barley	*Hordeum vulgare*	5100 Mb; 454, Bacterial Artificial Chromosome (BAC-by-BAC)	[26,75]
Finger millet	*Eleusine coracana*	1200 Mb; Illumina, Whole-Genome Sequencing (WGS)	[76,77]
Pearl millet	*Cenchrus americanus*	1800 Mb; Illumina, Whole-Genome Sequencing (WGS)	[25]
Proso millet	*Panicum miliaceum*	923 Mb; Illumina short-read coupled with Pac-Bio long-read sequencing	[78]
Barnyard millet	*Echinochloa esculenta*	1.27 Mb; Illumina HiSeq platform	[79]

## 3. Genomic Resources and Their Implications

**Figure 3:**
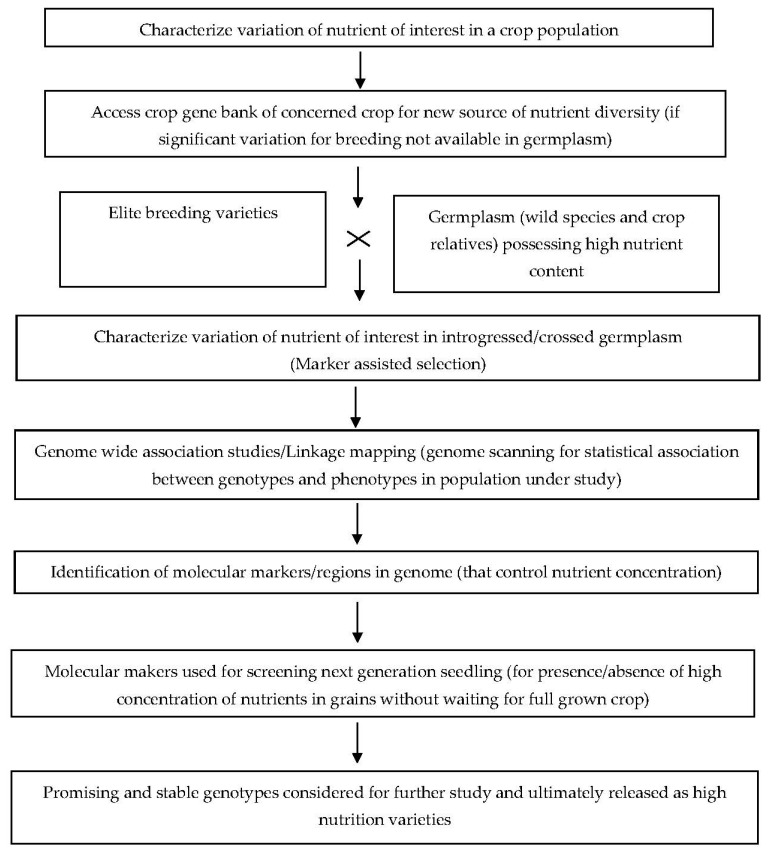
A prototype of marker-assisted introgression/selection for high nutrition varieties in crops.

Genomic resources play a crucial role in characterizing essential genes within a species, employing diverse approaches including structural and functional analyses, QTL/linkage mapping, and gene editing. These techniques have facilitated the genetic enhancement of cereal crops by deciphering complex trait architectures. Utilizing genomic resources in molecular breeding programs has effectively amplified cereal grain yield and crop productivity. Additionally, these resources optimize the utilization of genetic assets across different crop varieties (Figure 3).

Genomic resources have led to the identification and tagging of novel molecular markers through genome-wide association studies (GWAS), linking them to valuable agronomic and physiological traits. Furthermore, QTL mapping has become an instrumental approach for marker-assisted breeding (MAB), facilitating trait improvement and introgression. The downstream application of developed genomic resources has empowered plant breeders to introduce pivotal genes into various crops, particularly cereals. Notably, genomic resources have revolutionized the identification of significant genes/QTLs and their integration into diverse cereal crops.

This integration of crucial genes/QTLs has resulted in improved iterations of crop varieties that exhibit tolerance to environmental stresses. As a consequence, these enhanced varieties boast augmented yields and nutritional value. This substantial genetic progress is evident in crops such as rice, wheat, maize, and barley. In sum, genomic resources have significantly contributed to the genetic enhancement of multiple cereal crop traits [80,81].

In harnessing the genetic diversity present in cereal crop germplasm, genomic resources have played a pivotal role. For instance, in breeding programs, the inclusion of diverse genotypes in crossings is essential. Genomic resources prove invaluable in characterizing and identifying diverse parent candidates at the molecular level. Among various molecular breeding strategies, genomic selection (GS) has emerged as particularly fitting for improving intricate traits [82]. GS quantifies the collective impact of genome-wide markers to estimate the cumulative effects of all loci, yielding genomic estimated breeding values (GEBVs) that facilitate precise selection [83,84]. GS effectively captures alleles/QTLs governing traits with modest, heritable effects [85,86].

The following examples illustrate the deployment of essential genes/QTLs in grain cereal crops through methods such as MABC/MABB and GS, utilizing an array of genomic resources (Table 2).

Genomic resources have been pivotal in enhancing important agronomic traits via MABB. Examples include the *TaSUS2-2B* and *TaZds-D1* genes impacting grain weight [81], the Rht1 and Rht2 semi-dwarfing genes in wheat germplasm [115], and genes like *Lr34*, *Yr18*, *Pm38*, and *Sr45* enhancing disease resistance to rust and powdery mildew in wheat [118,119,120]. Additionally, genes like *Psy1*, *Glu-B3*, and *GS3* influence traits such as yellow grain pigment, low gluten protein, and longitudinal grain elongation, respectively [121,122,123]. Similarly, *Wx* gene encoded intermediate amylose content in rice [124], *Dwarf 8* gene for reduced plant height and early flowering in maize [125], and the *sugary1* gene for sweetness in maize [126]. Other instances encompass genes for bacterial blight resistance and sweetness in rice, as well as genes governing traits in maize and barley [84,85,86].

The availability of genomic resources is pivotal for enhancing cereal and broader crop improvement endeavors. These resources not only facilitate the transfer of vital genes through molecular breeding, but also support studies on genetic diversity, genotype identification, population structure, and phylogenetics [127,128]. Multiple breeding approaches, including MAS, MABB, MARC, and GS, are leveraged for enhancing crop varieties to resist biotic and abiotic stresses, along with improving quality traits. The integration of genes/QTLs into diverse crop species through genomic resources has spawned the development and commercialization of various crop varieties, such as Improved Pusa Basmati 1, Improved Samba Mahsuri, Pusa 1121, Improved Tapaswini, and Ranbir Basmati [84,129,130,131,132]. Furthermore, numerous rice varieties and hybrid parental lines have been improved for resistance to bacterial blight (*xa5*, *xa13*, and *Xa21*), either independently or combined with genes/QTLs for other stresses and quality attributes [133].

## 4. Conclusions and Future Perspectives

The accessibility of genomic resources in conjunction with genotyping platforms has emerged as a cornerstone in advancing cereal crop varieties. Plant breeders are now empowered to unlock the genetic potential of cereal crop varieties by orchestrating native genes in various permutations through the utilization of diverse genomic resources. While molecular breeding techniques have been effectively harnessed for prominent cereal crops like rice, wheat, maize, and barley, considerable potential remains untapped for lesser-explored cereal varieties, such as sorghum, finger millet, and foxtail millet. A crucial avenue lies in establishing diverse bi-parental mapping populations to identify marker–trait associations in these underexplored crops.

In the future, the evolution of high-throughput genotyping and phenotyping techniques promises a comprehensive understanding of gene function and marker–trait correlations, enriching our capacity to further refine crop varieties. The advancement in genomic resources has revolutionized the enhancement of complex traits, a task previously arduous. Single-nucleotide polymorphism (SNP) markers have emerged as the preferred molecular markers, and the conversion of identified SNPs to cleaved amplified polymorphic sequences (CAPs) or KASPar assays, along with Illumina VeraCode, is poised to expand their application in cereal crop improvement programs.

The prospects for developing a plethora of genomic resources remain promising. The sequencing of genomes within and across species holds the potential to yield numerous novel resources. As we look ahead, the genetic enhancement of cereal crop varieties can be propelled through the integration of superior haplotypes into these varieties. Haplotype-based breeding approaches can pave the way for further advancements in cereal crop improvement.

In summary, the availability and utilization of genomic resources, coupled with innovative genotyping technologies, have ushered in a new era of cereal crop breeding. These resources not only refine existing crops, but also harbor the potential to rejuvenate neglected cereal varieties. As we continue to delve deeper into the realm of genomics, the canvas for cereal crop enhancement expands, promising increased yield, resilience, and nutritional value to meet the challenges of a growing global population and a changing environment.

## Figures and Tables

**Table 2:** Genomic resources and genotyping platforms for the improvement of major cereal crops.

Crop	Molecular Breeding Approaches	Traits Improved	References
Wheat	QTL mapping and GWAS using Infinium 90 K SNP assay	Drought tolerance	[87]
	GWAS using GBS	Spot blotch resistance	[88]
	GWAS using Illumina Infinium 15 K BeadChip	Head blast resistance	[89]
	GWAS using 35 K axiom^®^ arrays	Stripe rust resistance	[90]
	GWAS using DArTseq technology	Tan spot resistance	[91]
	GWAS using wheat 660 K SNP array	Identification of chromosomal regions of root traits	[92]
	Haplotype-based GWAS and genotyping by sequencing	Grain yield	[93]
	QTL mapping (RIL population derived from specific locus amplified fragment sequencing (SLAF-seq))	Grain weight and size	[94]
	GWAS using 20 K Infinitum iSelect SNP array and genomic prediction of anther extrusion in hybrid breeding	Helps in rapid breeding for a particular trait	[95]
	Marker-assisted selection	Pyramided genes of high grain weight, stripe rust, and leaf rust resistance	[96]
Rice	QTL mapping	Heat tolerance	[97]
	QTL mapping	Pre-harvest sprouting resistance	[98]
	SNP genotyping array RiceSNP50	Functional genomics and molecular breeding	[99]
	580 K SNP array	Genomic selection and GWAS	[100]
	Genotyping-by-sequencing	Pre-breeding and improvement	[101]
	Genomic prediction	Rice improvement	[102]
	GWAS	Adaptability to dry direct-seeded rice (DDSR) system	[103]
	Rice pangenome genotyping array (RPGA) SNP genotyping	Genetic improvement	[104]
	QTL-seq using NIL-F_2_	Grain length and weight	[103]
	SNP-based QTL mapping	Panicle traits	[104]
Maize	QTL mapping	Northern corn leaf blight resistance	[105]
	GWAS	Striga resistance	[106]
	DArT seq SNP genotyping	Striga resistance hybrid breeding	[107]
	5.5 K SNPs using genotyping by target sequencing (GBTS)	Genomic prediction	[108]
	GBS, 40 K SNP array and target sequence capture	Genomic prediction	[109]
	QTL mapping	Prolificacy trait	[110]
	Meta-QTL analysis	Fungal disease resistance	[111]
	Marker-assisted recurrent selection (MARS)	Grain yield	[112]
	GWAS	Ear rot resistance	[113]
	QTL/genomic region identification	Biotic stress resistance	[114]
Barley	*LTP2* through marker-assisted recurrent selection (MARS)	Semi-dwarf	[115]
	Introgression of *rpg4/Rpg5* gene through marker-assisted recurrent selection (MABB)	Stem rust disease resistance	[116]
Pearl millet	Introgression of *qRSg1* and *qRSg4* genes through marker-assisted selection (MAS)	Downy mildew disease resistance	[117]

## Data Availability

All data has been included in the manuscript.
